# Plasma Functionalization of Carbon-Based Materials for Electrocatalytic Applications

**DOI:** 10.3390/ma19173782

**Published:** 2026-09-05

**Authors:** Julia Wieczorek, Diego Ramón Lobato Peralta, Paweł Stelmachowski

**Affiliations:** 1 Faculty of Chemistry, Jagiellonian University, Gronostajowa 2, 30-387 Krakow, Poland; 2 Instituto de Carboquímica—Spanish National Research Council, Miguel Luesma Castán 4, 50018 Zaragoza, Spain; dlobato@icb.csic.es

**Keywords:** plasma functionalization, carbon-based electrocatalysts, defect engineering, heteroatom doping, surface modification, electrocatalysis

## Abstract

Carbon-based materials are widely employed in electrocatalytic energy conversion and storage technologies owing to their high electrical conductivity, chemical stability, tunable structure, and low cost. However, the limited intrinsic activity and surface inertness of pristine carbon materials often necessitate surface modification to generate catalytically active sites and improve interactions with reactants and electrolytes. Among the available approaches, plasma functionalization has emerged as a versatile, rapid, solvent-free, and potentially resource-efficient technique that enables systematic tuning of surface chemistry while often limiting modification primarily to the near-surface region. This review discusses the fundamentals of plasma-assisted surface modification of carbon materials, including plasma generation, reactive species, plasma–surface interaction mechanisms, and the influence of key processing parameters such as gas composition, power, pressure, and treatment time. Particular attention is devoted to plasma-induced heteroatom doping, defect engineering, surface functionalization, and the dynamic structural evolution of carbon frameworks during treatment. The impact of these modifications on the physicochemical properties and electrocatalytic performance of carbon materials is critically examined with respect to representative reactions, including the oxygen reduction, oxygen evolution, and hydrogen evolution reactions. The advantages, limitations, and scalability of plasma technologies are also discussed, along with current challenges in process control and reproducibility. Finally, future opportunities involving operando diagnostics, single-atom catalysts, advanced porous carbon architectures, and industrial-scale plasma processing are highlighted. Plasma processing offers a versatile route to carbon surface and catalyst-interface engineering, although standardized reporting and quantitative plasma–structure–performance relationships are still required for rational process design and scale-up.

## 1. Introduction

### 1.1. Role of Carbon Materials in Electrocatalysis

**Figure 1:**
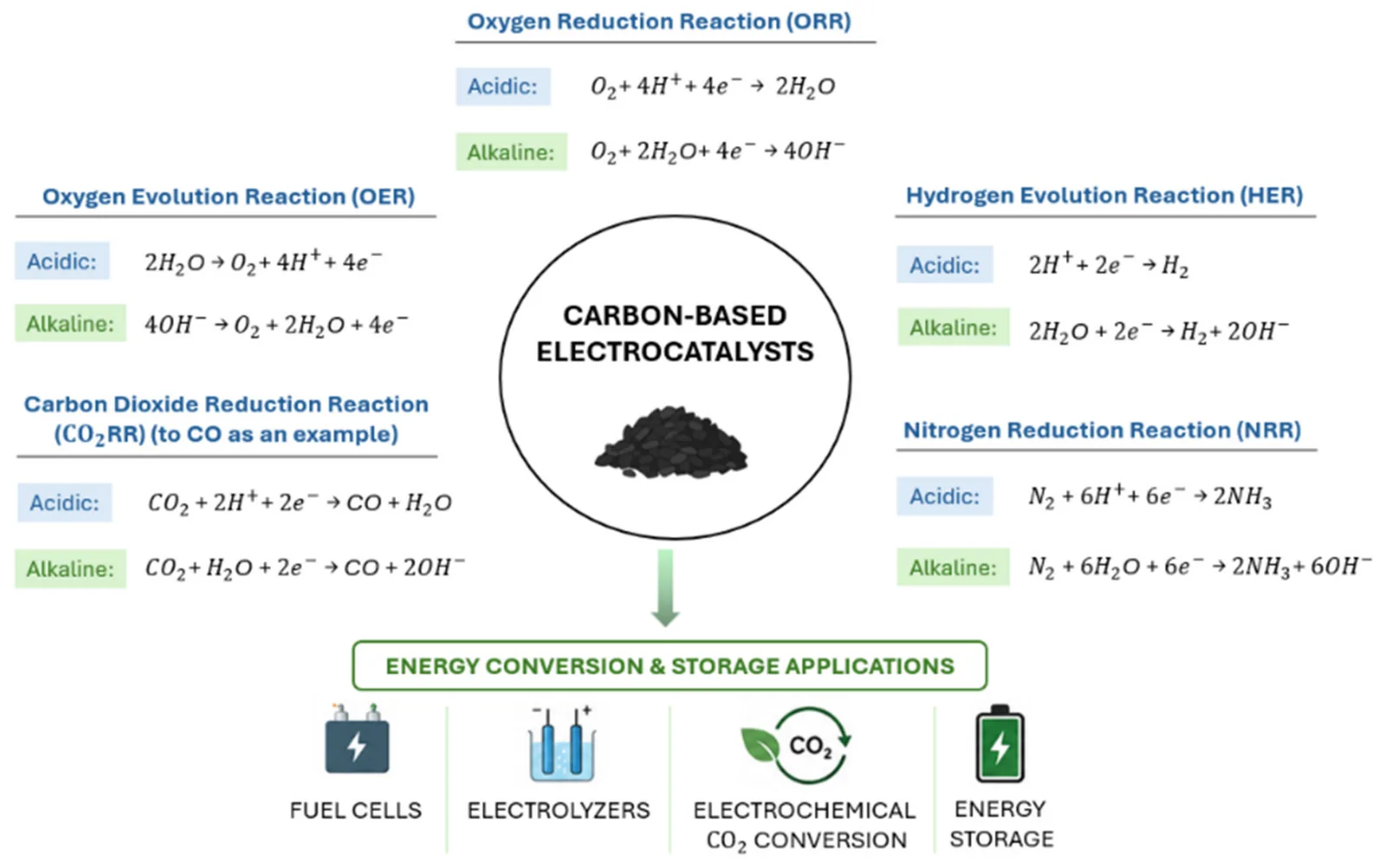
Schematic overview of the main electrocatalytic reactions involving carbon-based materials and their representative energy conversion and storage applications.

Electrocatalysis plays a fundamental role in energy conversion and storage technologies by accelerating electrochemical reactions at electrode surfaces and improving their efficiency. The development of effective electrocatalysts is essential for applications such as fuel cells, electrolyzers, and electrochemical carbon dioxide conversion. Among the various catalyst materials, carbon-based materials have attracted considerable attention due to their exceptional physicochemical properties [1,2,3]. They are employed in key electrocatalytic processes, including the oxygen reduction reaction (ORR), oxygen evolution reaction (OER), and hydrogen evolution reaction (HER), as well as the carbon dioxide reduction reaction (CO_2_RR), and nitrogen reduction reaction (NRR) [4,5]. A schematic overview of the major electrocatalytic reactions involving carbon-based materials and their applications in energy conversion and storage technologies is presented in Figure 1. These processes highlight the pivotal role of carbon materials in sustainable electrocatalytic applications.

The advantages of carbon materials arise from several key factors that enhance their electrocatalytic performance. Their high electrical conductivity facilitates efficient electron transport and accelerates reaction kinetics [1,6,7]. Carbon materials possess excellent chemical and mechanical stability, enabling operation under demanding electrochemical conditions. Their structure and surface properties can be readily tailored through defect generation, edge-site exposure, and heteroatom doping, thereby creating additional active sites and tuning adsorption characteristics [8,9,10].

Despite these benefits, pristine carbon materials possess inherent limitations. Their chemical inertness and hydrophobic nature can reduce interactions with aqueous electrolytes and limit intrinsic catalytic activity, highlighting the need for surface modification or functionalization to enhance performance [6]. Approaches developed to overcome these limitations can be broadly divided into conventional surface functionalization methods and plasma-based modification strategies, which are discussed in Section 1.2 and Section 1.3, respectively.

### 1.2. Surface Functionalization Strategies: A Brief Overview

Surface functionalization is a critical step in tailoring the physicochemical properties of carbon materials for specific applications, such as adsorption and catalysis. To achieve this, various strategies have been developed over the years. Conventional surface functionalization methods include wet chemical oxidation, thermal annealing, and electrochemical activation [11].

Wet chemical oxidation introduces oxygen-containing functional groups, such as hydroxyl and carboxyl groups, onto the carbon surface via treatment with oxidizing reagents. This process enhances the material’s hydrophilicity and creates reactive surface sites [12]. However, the method often suffers from limited selectivity and may adversely affect the structural properties of carbon materials. For example, wet oxidation treatments have been reported to cause shortening of carbon nanotubes (CNTs), damage to CNTs sidewalls, and reductions in the surface area and total pore volume of activated carbons [13,14].

Thermal annealing is a widely used heat-treatment method employed to modify the properties of various materials, including metals, ceramics, semiconductors, and carbon-based materials. The process involves exposing a material to elevated temperatures under a controlled atmosphere, which can lead to the removal of structural defects, changes in surface chemistry, and improved structural ordering [15,16,17,18]. However, the outcome of annealing strongly depends on process parameters such as temperature, treatment time, and atmosphere. For example, in the case of single-walled carbon nanotubes (SWCNTs), an optimal annealing temperature of approximately 400 °C has been reported, whereas higher temperatures result in the generation of additional structural defects and deterioration of the material properties [18].

Electrochemical activation is a process that employs an electric field or current flow to modify the surface properties of materials and generate reactive chemical species. It is commonly applied to materials such as carbon electrodes, metal oxides, and biochar to enhance their surface activity, electrical conductivity, and adsorption capacity. The reactive species generated during the process, including free radicals, can participate in oxidation reactions, for example, during the degradation of pollutants in wastewater [19,20]. Despite its advantages, the efficiency of electrochemical activation may be limited by side reactions. One example is the OER during the electrochemical oxidation of methane, which reduces process selectivity [21]. Another challenge is the lack of standardized experimental protocols, particularly in studies involving the activation of three-dimensional electrode surfaces [22]. Process parameters are often selected based on individual experimental results without adequate statistical validation, making it difficult to reproduce and compare findings across different studies. These limitations have motivated the development of alternative techniques capable of achieving more precise surface-specific functionalization [23,24].

### 1.3. Plasma Functionalization: Key Advantages

Plasma-based techniques, widely used for surface modification of materials, represent a versatile approach to carbon functionalization. In plasma processes, highly reactive species, including ions, radicals, and excited atoms or molecules, as well as ultraviolet irradiation, interact with the material surface, inducing controlled physicochemical transformations. Unlike traditional methods, plasma treatment is solvent-free, rapid, and can be performed at relatively low temperatures, enabling surface modification while limiting extensive thermal damage to the material [25].

An important advantage of plasma functionalization is the ability to tailor surface properties by adjusting process parameters such as gas composition, power, pressure, and treatment time. Depending on the processing conditions, plasma treatment may promote the formation of functional groups, defect sites, and heteroatom-containing surface species. Because plasma-material interactions are primarily confined to the near-surface region, surface chemistry can be modified while often maintaining the bulk characteristics of the material [26].

Plasma treatment has been successfully applied to a wide range of carbon materials; however, the extent and uniformity of modification depend strongly on reactor configuration, plasma characteristics, material morphology, and operating conditions. Owing to its flexibility and broad applicability, plasma processing has attracted considerable interest as a tool for engineering carbon-based electrocatalysts with tailored physicochemical properties [27].

Despite its advantages, plasma functionalization also presents several challenges, including treatment non-uniformity, limited penetration into porous materials, scale-up difficulties, and the need for advanced process diagnostics and control [28].

**Figure 2:**
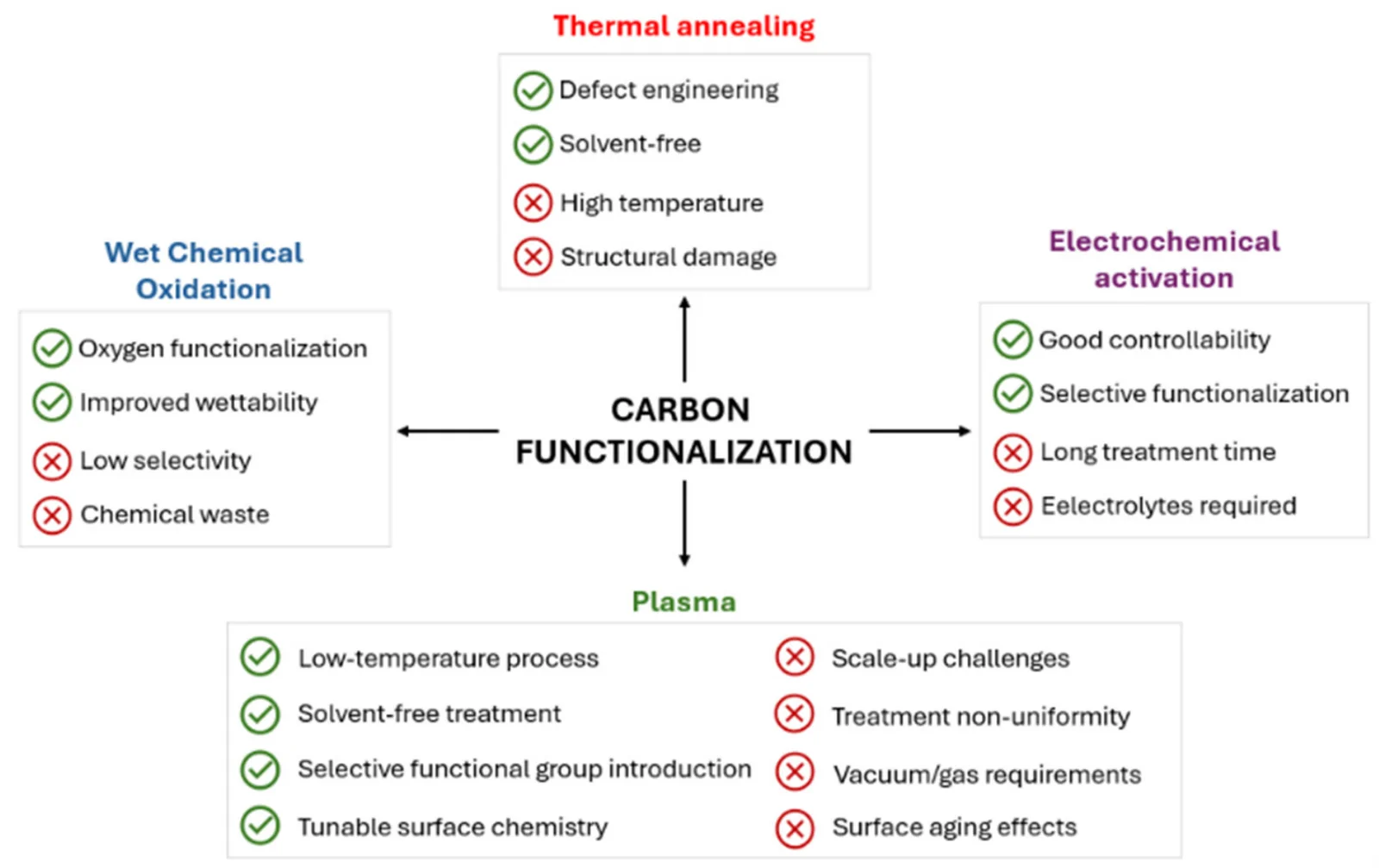
Schematic comparison of the principal carbon functionalization methods, including wet chemical oxidation, thermal annealing, electrochemical activation, and plasma functionalization. The main advantages and limitations of each approach are summarized.

Figure 2 presents a schematic comparison of the conventional carbon functionalization methods discussed in Section 1.2 and the plasma-based functionalization approach presented in Section 1.3, highlighting their selected advantages and limitations.

Previous reviews have examined non-thermal plasma synthesis and surface modification across broad classes of electrocatalysts and electrode materials, including noble-metal, non-noble-metal, and metal-free systems for fuel cells, water splitting, batteries, and supercapacitors [5,29]. More recent reviews have focused either on an individual electrocatalytic reaction, particularly OER [30], or on a specific class of plasma systems without a dedicated electrocatalytic focus, such as atmospheric-pressure plasma processing of carbon materials [28]. However, a critical comparison focused specifically on the relationships among plasma-processing conditions, modification of carbon structures and interfaces, and reaction-specific electrocatalytic performance remains limited. The present review addresses this gap by distinguishing direct carbon functionalization, plasma-assisted synthesis of carbon-containing catalysts, and plasma treatment of complete carbon-supported electrode architectures. Particular emphasis is placed on quantitative processing and electrochemical parameters, the limitations of cross-study comparison, and the reporting standards required to establish reliable plasma–structure–performance relationships.

## 2. Fundamentals of Plasma Processing

Despite significant advances in the functionalization of carbon materials, achieving systematic tuning over the concentration and distribution of structural defects, the nature of functional groups, and electrochemical properties remains challenging [31]. Conventional approaches often face limitations in simultaneously ensuring selectivity, uniformity, and preservation of the material structure, thereby restricting their applicability in advanced electrocatalytic systems [6,32,33].

To fully exploit the potential of plasma functionalization, it is essential to understand the fundamental principles governing plasma generation and plasma–surface interactions. Plasma contains high-energy electrons, ions, radicals, and excited species that can induce a wide range of physical and chemical processes at the material surface. The nature and extent of these interactions depend strongly on the plasma characteristics and operating conditions, which ultimately determine the structural and chemical modifications introduced into carbon materials. A fundamental understanding of the relationships among plasma type, generated species, and process parameters is therefore key to the rational design of plasma-assisted surface modification processes [28]. These factors govern the formation of defects, the incorporation of surface functionalities, and the evolution of surface composition, thereby influencing the activity, selectivity, and durability of carbon-based electrocatalysts [28,34,35,36].

### 2.1. Types of Plasmas Relevant to Materials Modification

The type of plasma employed determines the nature of the reactive species generated and the mechanisms governing their interaction with the material surface. Consequently, the control of defect density and surface chemistry is strongly dependent on process parameters associated with the plasma source and operating conditions [28,37,38]. Among the various plasma types, low-pressure radio-frequency (RF) plasma plays a particularly important role in surface modification of materials, alongside microwave and inductively coupled plasma (ICP) systems, as well as atmospheric-pressure plasmas such as dielectric barrier discharge (DBD) and plasma jets. The main differences between low-pressure and atmospheric-pressure plasma systems, including their characteristic reactive species, dominant mechanisms, and typical effects on carbon materials, are summarized in Table 1.

Low-pressure RF plasma is widely used for surface modification due to its high degree of process controllability [39,40]. Typically operated at 13.56 MHz, RF plasma systems can provide control over ion energy and plasma density; however, independent adjustment of these parameters generally requires additional biasing, dual-frequency excitation, or advanced plasma configurations such as ICP systems. Under low-pressure conditions, a non-equilibrium plasma is established, where the electron temperature significantly exceeds the gas temperature, enabling efficient ionization and dissociation of precursor species [39]. Consequently, a flux of reactive species, including ions, radicals, and excited neutrals, is generated. Their interaction with the surface involves both physical (ion bombardment) and chemical pathways, enabling defect formation and surface functionalization under controlled processing conditions while often limiting modifications primarily to the near-surface region of the material [41].

Microwave plasmas and ICP systems are characterized by significantly higher plasma densities than other plasma system types discussed above. Microwave plasmas, typically generated at 2.45 GHz, allow electrode-less operation, thereby reducing contamination and improving plasma uniformity [42]. In ICP systems, the discharge is sustained by electromagnetic induction, resulting in high plasma densities at relatively low ion energies. The decoupling of these two parameters is advantageous in processes requiring high concentrations of reactive species while minimizing surface damage [43].

Atmospheric-pressure plasmas, including DBD and plasma jets, offer an alternative by eliminating the need for vacuum systems. This makes them attractive for large-scale and industrial applications. Although these systems generally provide less systematic tuning over ion energy, they generate high densities of reactive species and are effective for surface activation, cleaning, and functionalization [44]. Plasma jets enable localized treatment through directed delivery of reactive species, whereas DBD systems are suitable for large-area surface treatment; however, the uniformity of modification depends strongly on the discharge mode and operating conditions, as many atmospheric-pressure DBD systems exhibit filamentary behavior [45].

Overall, the choice of plasma system strongly influences the resulting surface chemistry and defect structure. These differences arise from variations in plasma density, ion energy, operating pressure, and the nature of reactive species. Microwave and ICP systems favor high reaction rates, whereas atmospheric-pressure plasmas offer practical advantages in terms of scalability [45,46,47].

### 2.2. Reactive Species and Surface Interaction Mechanisms

Plasma constitutes a highly reactive environment containing a mixture of charged and neutral species, including electrons, positive and negative ions, radicals, metastable excited species, and electromagnetic radiation, particularly in the ultraviolet (UV) range. These components collectively govern the nature and extent of interactions occurring at the plasma-material interface [48].

Among these, free radicals play a particularly important role due to their high chemical reactivity and relatively high flux in low-temperature plasmas. They are generated by electron-induced dissociation of the process gas, and their nature strongly depends on the plasma composition. Consequently, oxygen-, nitrogen-, or hydrogen-containing plasmas generate corresponding reactive radical species [49]. Under such conditions, the flux of neutral radicals typically exceeds that of ions, so surface reactions are predominantly governed by radical-driven chemical pathways. These species readily participate in addition and substitution reactions, leading to the incorporation of functional groups and modification of surface chemistry [48,50]. In contrast, ions contribute primarily through physical interactions, such as ion bombardment, which induces sputtering, surface activation, and bond scission. Ion impacts can further enhance surface reactivity by generating dangling bonds and facilitating subsequent reactions with neutral species. Metastable species and UV photons also contribute to these processes by transferring energy, inducing electronic excitation, and promoting dissociation processes without direct particle impact [51].

The balance between physical (ion-induced) and chemical (radical-driven) mechanisms determines whether plasma treatment results predominantly in material removal (etching) or surface functionalization [52]. This balance is strongly dependent on plasma parameters, including pressure, input power, gas composition, and treatment time, which govern the energy and flux of plasma species [53]. Collectively, these plasma-induced processes alter the defect structure, surface energy, and electronic properties of the material. In carbon-based electrocatalysts, they play a critical role in controlling the density and nature of active sites, thereby influencing charge transfer, adsorption behavior, and overall catalytic performance [54].

### 2.3. Controllable Parameters and Their Effect

Plasma-assisted surface modification of carbon materials is an effective approach whose efficiency strongly depends on operating conditions. Parameters such as gas composition, discharge power, operating pressure, substrate bias voltage, and treatment duration influence the concentration, energy, and reactivity of the species generated within the plasma phase [55,56]. Systematic tuning of these variables is crucial for tailoring the concentration and distribution of defects, surface chemistry, and the electrical properties of carbon materials intended for electrocatalytic applications [57,58,59].

Plasma modification may introduce functional groups and increase the number of active sites on the surface, thereby improving the electrocatalytic performance of the modified materials [55]. Plasma-induced defect engineering plays a key role in enhancing catalytic activity by altering the electronic structure and physicochemical characteristics of the carbon framework. These structural and chemical changes influence mass and charge transfer dynamics, active-site exposure, and product selectivity in electrocatalytic reactions [58].

Depending on the severity of the treatment and operating conditions, plasma processing may predominantly result in surface cleaning, functionalization, or etching. The relative contribution of these processes is governed by the balance between physical and chemical interactions occurring at the plasma–surface interface [60]. These effects and their underlying mechanisms are discussed in greater detail in the following sections.

**Figure 3:**
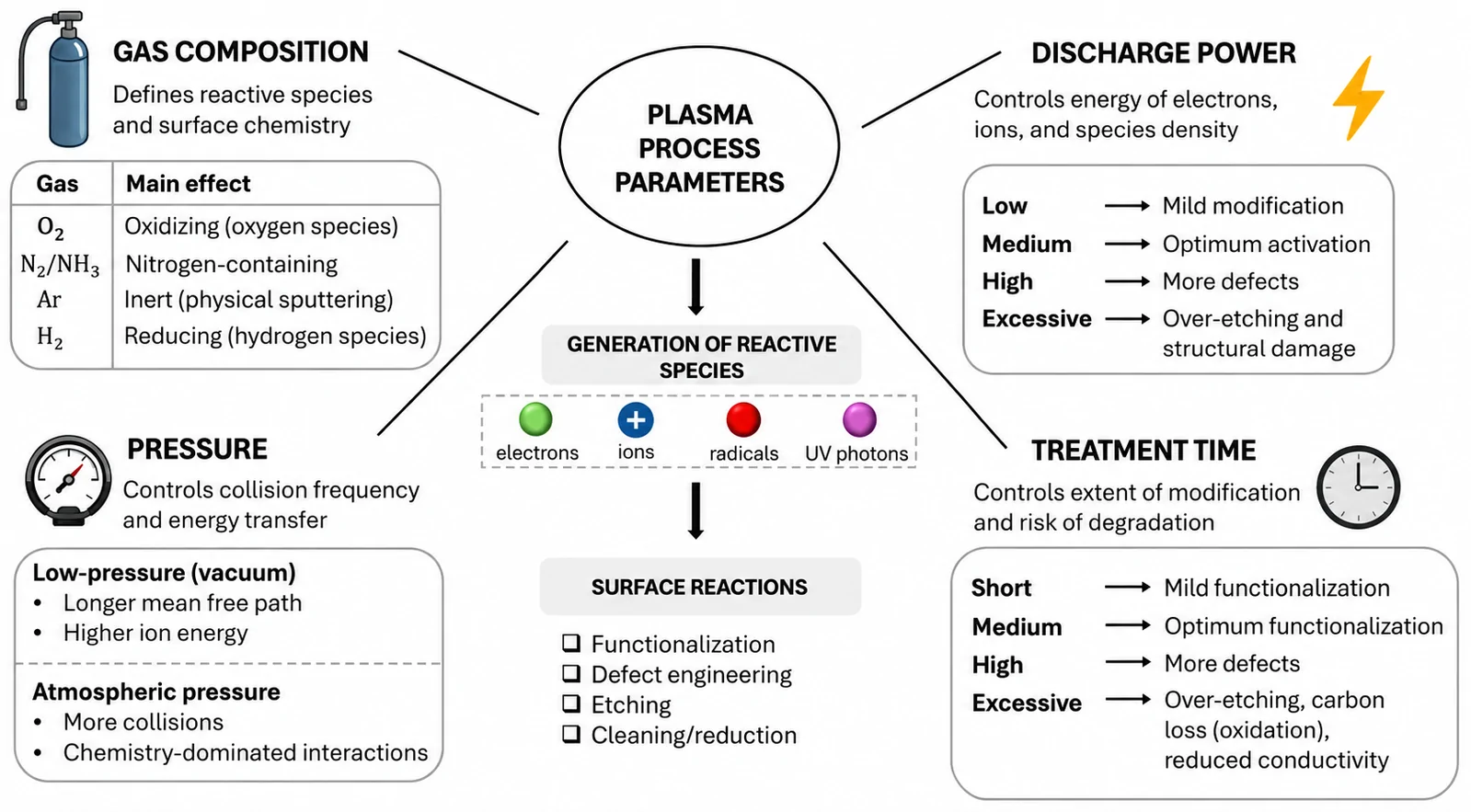
Schematic illustration of the relationship between plasma process parameters, reactive species generation, and the resulting surface modification mechanisms.

As summarized in Figure 3, plasma process parameters determine the type, concentration, and energy of reactive species generated in the discharge, thereby governing the balance between surface functionalization, defect formation, cleaning, and etching.

Reliable interpretation of plasma functionalization requires complementary plasma diagnostics and material characterization. Optical emission spectroscopy and mass spectrometry can identify excited and stable gas-phase species, while electrical measurements, Langmuir probes where applicable, ion-energy analysis, and substrate-temperature monitoring provide information beyond nominal generator settings. The resulting carbon modification should be evaluated by combining X-ray Photoelectron Spectroscopy (XPS) and elemental analysis for surface and bulk composition, Raman spectroscopy and Electron Paramagnetic Resonance (EPR) for structural and paramagnetic defects, electron microscopy and Brunauer–Emmett–Teller (BET) analysis for morphology and porosity, contact-angle measurements for wettability, and Ultraviolet Photoelectron Spectroscopy (UPS) or work-function measurements for electronic changes. Operando or post-reaction spectroscopy is additionally required to identify reconstruction, leaching, and ageing. No single technique can independently distinguish doping, oxidation, defect generation, pore development, and active-phase redistribution.

#### 2.3.1. Gas Composition

Among the aforementioned factors, gas composition is particularly important because it determines the type of reactive species generated during the plasma discharge. Oxygen-containing plasmas promote oxidation processes and the incorporation of oxygen-containing functional groups, such as hydroxyl, carbonyl, carboxyl, and epoxy groups [26,61,62,63].

For example, oxygen plasma treatment may lead to the decoration of graphene surfaces with -OH groups [62] and the introduction of high-energy oxygen-containing functionalities into carbon-fiber composites [64]. In contrast, nitrogen-containing plasmas are widely used for heteroatom doping, enabling the incorporation of pyridinic, pyrrolic, and graphitic nitrogen configurations into the carbon structure [65,66]. These modifications significantly influence the electronic structure and catalytic activity of carbon-based materials. For example, pyridinic and graphitic nitrogen species may activate adjacent carbon atoms, thereby enhancing electrocatalytic performance [67].

Hydrogen plasmas, in turn, are commonly used for surface cleaning, reduction of oxygen-containing groups, and selective etching processes. Under appropriate plasma conditions, hydrogen treatment may lead to hydrogen termination of the surface and the formation of hydrogenated amorphous carbon-like layers, which provide lubrication and reduce adhesive wear [68,69].

Plasma systems, including multicomponent plasma environments, enable both functionalization and defect generation in carbon materials. Such plasma environments enable simultaneous defect engineering and heteroatom incorporation without substantial modification of the bulk carbon structure [70]. Appropriate selection of process parameters may also enable the selective promotion of specific heteroatom configurations, such as pyridinic or graphitic nitrogen, which exhibit distinct electrocatalytic properties. For example, graphitic nitrogen coordinated with pentagonal defects may significantly enhance electrocatalytic activity [66]. Different nitrogen configurations, including pyridinic and graphitic nitrogen, play distinct roles in electrocatalytic reactions, such as oxygen reduction, influencing the selectivity of reaction pathways [71].

#### 2.3.2. Discharge Power

Discharge power and operating pressure significantly influence plasma density [72,73], electron temperature [74], as well as the ion energy distribution and the frequency of ion-neutral collisions [75]. Increasing plasma power generally enhances ionization and dissociation, resulting in higher concentrations of radicals and ions that can interact with the material surface [76,77]. Consequently, higher power promotes the generation of more defects and surface activation [64]. However, excessive power may lead to structural degradation, over-etching, and severe disruption of graphitic ordering [78].

#### 2.3.3. Pressure

Pressure influences the collision frequency and the energy-transfer processes within the plasma [79]. Low-pressure systems typically provide higher-energy ion bombardment and greater control over surface processes [80], whereas atmospheric-pressure plasmas can generate high densities of reactive species, although their interactions are generally more chemical than physical in nature [81,82].

#### 2.3.4. Time

Treatment duration is another important parameter that determines the extent of surface modification [83,84]. Short exposure times typically result in mild functionalization and limited defect generation, whereas prolonged treatment increases the concentration of functional groups and the density of structural defects. However, excessively long processing times may lead to over-etching, carbon loss due to oxidation, and degradation of the conjugated π-electron system, resulting in reduced electrical conductivity and hindered charge transport [84]. In many cases, the relationship between treatment duration and catalytic activity is non-linear, with optimal performance observed for materials subjected to moderate levels of functionalization [78].

Overall, plasma process parameters determine the balance between surface activation, heteroatom doping, defect generation, and preservation of the ordered graphitic structure. Rational optimization of these parameters is crucial for designing carbon materials with controlled surface chemistry, appropriate electronic properties, and high electrocatalytic activity [85]. It should be emphasized, however, that plasma process parameters remain strongly interdependent, and the final effect of surface modification results from a balance among etching, functionalization, and structural reconstruction. Consequently, optimizing the process parameters requires a compromise between intensive surface activation and the preservation of the material’s structural integrity and high electrical conductivity [10]. Ultimately, the optimal combinations of plasma treatment parameters depend on the specific device and must therefore be determined experimentally. The resulting structural and chemical modifications of carbon materials are discussed in the following section.

## 3. Plasma–Carbon Interactions: Mechanisms and Structural Evolution

### 3.1. Interaction with sp^2^ and sp^3^ Carbons

The interaction of plasma with carbon-based materials is largely governed by the hybridization state of carbon atoms, primarily sp^2^ and sp^3^ configurations. In graphitic structures dominated by sp^2^ bonding, carbon atoms are arranged in planar hexagonal lattices with delocalized π electrons, which are responsible for high electrical conductivity and structural stability. In contrast, sp^3^-hybridized carbon exhibits a three-dimensional tetrahedral structure, in which electrons are more localized, resulting in lower electrical conductivity [86,87,88,89].

Plasma interaction leads to the dissociation of chemical bonds through a combination of physical and chemical mechanisms, the relative contributions of which are strongly dependent on the process conditions [90]. Energetic ion bombardment plays a dominant role in physical modification, leading to C-C bond scission, atomic displacement, and the formation of structural defects such as vacancies, dangling bonds, and non-hexagonal ring configurations [91]. These processes are often associated with increased disorder and partial amorphization of initially ordered sp^2^ domains [92].

A key aspect of carbon–plasma interactions is the possibility of interconversion between sp^2^ and sp^3^ hybridization states, which is highly sensitive to plasma parameters [88,93]. High-energy ion bombardment can induce the transformation from ordered sp^2^ domains to more disordered structures with increased sp^3^ character, whereas structural relaxation and reconstruction processes may promote the reverse transition [94]. Consequently, the carbon structure remains highly dynamic during plasma treatment and continuously evolves in response to changes in process parameters. For example, an increase in the sp^3^-bonded carbon fraction in amorphous carbon films has been observed with increasing ion energy and plasma density, demonstrating that the sp^3^/sp^2^ ratio can be tailored through appropriate control of the plasma parameters [95].

The extent and nature of plasma-induced modifications also strongly depend on the type of carbon material [96]. Graphitic structures exhibit relatively high resistance to bulk degradation and primarily react at edge sites, which are energetically favorable for chemical interactions [94]. In contrast, amorphous carbons are more susceptible to bond rearrangement and defect generation [97]. Porous carbon materials, due to their high specific surface area, provide enhanced accessibility for reactive plasma species, resulting in intensified plasma–surface interactions and more pronounced structural evolution [98].

**Figure 4:**
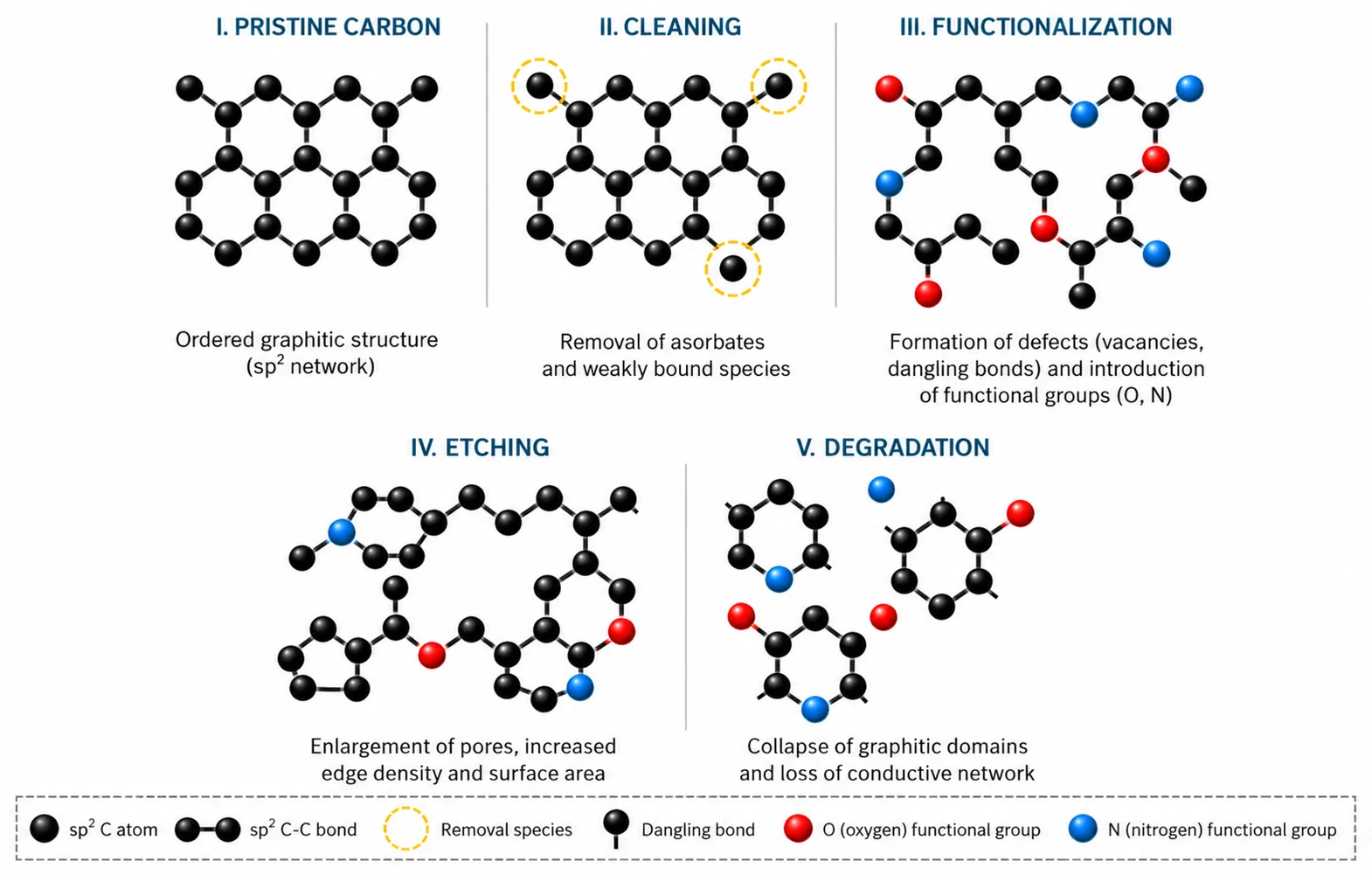
Schematic illustration of the progressive plasma-induced modification of graphitic carbon, including surface cleaning, functionalization, etching, and degradation.

Figure 4 summarizes the progressive structural evolution of carbon materials during plasma treatment, illustrating the transition from pristine carbon to surface cleaning, functionalization, etching, and, under excessive treatment conditions, degradation.

### 3.2. Surface Activation, Oxidation, and Etching

Plasma exposure induces substantial modifications in the surface chemistry and morphology of carbon materials through simultaneous activation, oxidation, and etching processes [28]. Due to the highly non-equilibrium nature of plasma, reactive species, including ions, radicals, excited molecules, metastable species, and UV photons, continuously interact with the carbon surface, initiating complex physicochemical transformations [99]. These interactions promote bond dissociation, defect generation, disruption of the conjugated sp^2^ network, and the formation of highly reactive surface sites, which significantly alter the electronic, catalytic, and adsorption properties of carbon materials [28,100,101].

In low-pressure plasmas, high-energy ion bombardment together with radical attack initiates localized bond scission, leading to the formation of vacancies, dangling bonds, topological defects, and distortion of graphitic domains [101,102]. Moderate plasma treatment may therefore introduce controlled structural disorder without destroying the conductive carbon framework. In graphitic materials, partial disruption of π-electron delocalization frequently results in fragmentation of ordered domains into smaller nanocrystalline regions, accompanied by increased structural disorder [103]. Such plasma-induced defect engineering is particularly important for catalytic applications because defective carbon sites often exhibit enhanced adsorption properties and modified charge distribution, which can facilitate electrochemical reactions [6]. An example of plasma-induced defect generation in carbon materials was reported by Hoque et al., who employed nitrogen plasma to modify graphitized amorphous carbon thin films. Raman spectroscopy revealed an increase in structural disorder associated with the formation of defects and progressive amorphization of graphitic domains. Initially, plasma treatment selectively etched the more disordered amorphous regions, whereas prolonged exposure gradually disrupted graphitized clusters. The authors demonstrated that increased defect density improved the electrocatalytic performance of the materials for the ORR, highlighting the important role of plasma-induced defects in tailoring the functional properties of carbon materials [104].

Simultaneously, plasma treatment often induces surface oxidation through reactions between reactive oxygen species and carbon atoms [27]. Depending on the plasma chemistry and processing conditions, oxygen-containing functional groups such as hydroxyl, carbonyl, epoxide, and carboxyl species may form on the carbon surface [101]. While oxygen-containing functional groups generally improve wettability and surface reactivity, excessive oxidation may disrupt the conjugated sp^2^ network and reduce electrical conductivity [6]. Moreover, excessive oxidation may destabilize the carbon framework and accelerate structural degradation [103].

Plasma-induced etching further contributes to the morphological evolution of carbon materials [101,105]. Reactive plasma species preferentially attack defect-rich and amorphous regions, whereas highly ordered graphitic domains generally exhibit greater resistance to plasma-induced erosion [101,103]. Under controlled processing conditions, selective plasma etching may lead to enlargement of existing nanopores, improved pore interconnectivity, and increased accessible surface area through the gradual removal of disordered carbon regions [105,106]. Such structural modifications are particularly beneficial for electrochemical systems because hierarchical porous architectures facilitate mass transport, electrolyte accessibility, and ion diffusion kinetics [6]. For example, plasma treatment of fibrous carbon materials has been shown to generate interconnected hierarchical pore networks that improve access to internal active sites and promote faster electrochemical kinetics [107].

However, prolonged plasma exposure or excessive ion bombardment may shift the balance from beneficial surface activation toward destructive structural degradation. Excessive etching can induce pore coalescence, thinning of carbon walls, collapse of porous frameworks, and significant deterioration of electrical conductivity due to progressive disruption of conductive sp^2^ domains [103,108,109]. Consequently, plasma processing requires careful optimization to balance controlled defect generation, pore development, and preservation of structural integrity [6,110].

### 3.3. Surface Relaxation and Reconstruction

The structure of plasma-modified carbon materials does not remain static after defect generation but undergoes further evolution associated with energetic relaxation and atomic reconstruction processes [101,102]. Defects formed during ion bombardment, such as vacancies, dangling bonds, and distortions of the graphitic lattice, possess high local energy and may undergo migration, recombination, or reorganization into more stable configurations [103]. As a result, the carbon surface remains highly dynamic even after direct plasma exposure, while the final structural state strongly depends on processing conditions, plasma chemistry, and the intrinsic properties of the carbon material [111].

Under specific conditions, particularly during mild plasma treatment, hydrogen-assisted plasma exposure, or elevated substrate temperatures, partial reconstruction of the ordered sp^2^ carbon network may occur through recombination of dangling bonds and local atomic rearrangement, leading to stabilization of graphitic domains and partial reduction in structural disorder [112,113]. These processes can promote the restoration of graphitic domains, the partial recovery of electron conjugation, and the reduction in structural disorder. In contrast, excessive plasma exposure or overly high ion energies may suppress relaxation pathways and instead promote further amorphization and irreversible structural damage [103,109].

Plasma-induced structural evolution is additionally governed by the competition between surface functionalization and etching processes [101]. During the initial stages of plasma treatment, reactive species may promote the incorporation of heteroatoms and the formation of oxygen- or nitrogen-containing functional groups, thereby enhancing surface activity and improving electrochemical properties [27]. Simultaneously, moderate plasma exposure increases the density of catalytically active defect sites without severely disrupting the conductive sp^2^ carbon framework. However, prolonged plasma treatment progressively enhances carbon removal rates and defect accumulation, eventually shifting the process from beneficial activation toward irreversible structural degradation [109]. Consequently, the balance between functionalization and etching strongly determines the final morphology, electronic structure, and physicochemical stability of plasma-modified carbon materials [101,108,114].

Another aspect of surface relaxation is the reactivity of carbon materials after plasma treatment is terminated. In particular, in vacuum systems, plasma-activated carbon surfaces undergo further reactions upon exposure to ambient air. The surface activation is such that plasma treatment with Ar can yield similar results to oxygen plasma treatment [115]. Furthermore, the degree of functionalization changes over time after plasma treatment, as evidenced by changes in electronic properties and surface oxygen content. These post-plasma effects can be used to further functionalize the surface by exposing it to various chemicals, and certainly must be taken into account in the electrocatalytic testing protocols [61].

The dynamic evolution of plasma-treated carbon structures has important implications for the long-term stability of carbon-based electrocatalysts [116]. Although defect-rich carbon frameworks frequently exhibit enhanced catalytic activity due to the increased density of active sites [6], excessive disorder may simultaneously accelerate electrochemical corrosion and structural degradation during prolonged operation [117]. Highly defective regions are generally more susceptible to oxidation, carbon loss, and conductivity deterioration under harsh electrochemical conditions [118]. Therefore, optimization of plasma processing conditions is essential to balance catalytic activity with long-term structural and electrochemical stability [119].

## 4. Plasma Engineering of Carbon Materials for Electrocatalysis

**Figure 5:**
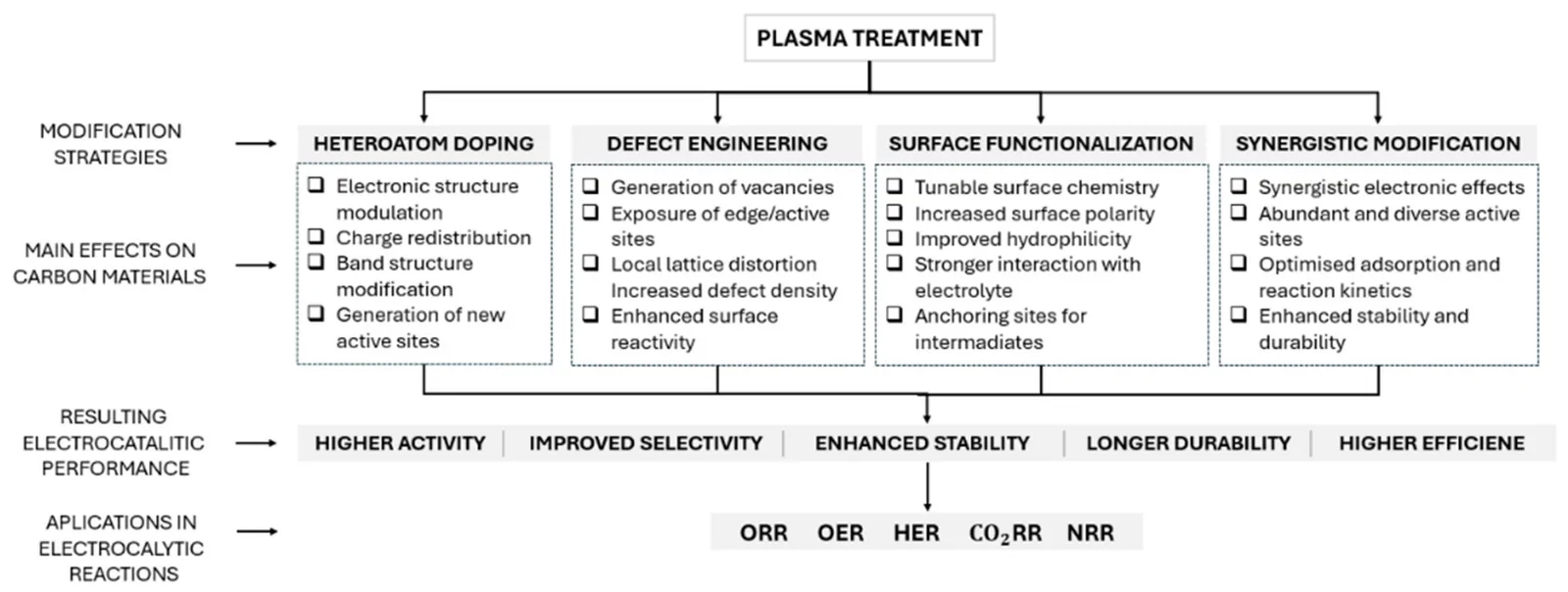
Schematic overview of plasma-induced modification strategies, their effects on the physicochemical properties of carbon materials, and their applications in representative electrocatalytic reactions.

The electrocatalytic activity of carbon materials is closely related to their electronic structure, surface chemistry, and the availability of catalytically active sites. These properties can be effectively tailored through plasma treatment, which enables heteroatom doping, defect generation, and surface functionalization. Accordingly, this chapter is divided into two sections: Section 4.1 reviews the principal plasma-engineering strategies applied to carbon materials and their effects on the physicochemical properties of these materials. In contrast, Section 4.2 discusses their application in representative electrocatalytic reactions. Figure 5 schematically summarizes these modification strategies, their influence on the physicochemical properties of carbon materials, and the resulting electrocatalytic applications.

### 4.1. Plasma-Induced Modification Strategies

#### 4.1.1. Heteroatom Doping

Heteroatom doping is one of the most widely employed strategies for tailoring carbon materials for electrocatalytic applications. Plasma treatment enables the incorporation of heteroatoms, such as nitrogen, sulfur, boron, and phosphorus, into the carbon framework while largely preserving the bulk structure and allowing systematic tuning rather than absolute control of dopant concentration and bonding configuration [120,121].

The incorporation of heteroatoms modifies the electronic properties of carbon materials. Differences in electronegativity and atomic size between carbon and the dopant atoms induce local redistribution of electron density and polarization of neighboring carbon atoms. As a result, new catalytically active sites are created, and the interactions between the catalyst surface and adsorbed reactants or reaction intermediates are altered [121].

Furthermore, the presence of heteroatoms may induce local distortion of the graphitic lattice and promote the formation of structural defects, thereby increasing the number of accessible active sites. At the same time, the extent of doping and defect formation depends strongly on plasma parameters such as gas composition, ion energy, and treatment duration, which influence the adsorption energy of key reaction intermediates and facilitate charge transfer. The overall effect of heteroatom doping depends on the type of dopant, its bonding configuration, concentration, and distribution within the carbon framework [122].

#### 4.1.2. Defect Engineering

Defect engineering is another key strategy for tailoring the physicochemical properties of carbon materials. Plasma treatment enables the controlled generation of structural defects, including vacancies, edge sites, dangling bonds, and topological defects, provided that the treatment severity is carefully adjusted to avoid excessive disruption of the carbon framework [123].

The introduction of defects modifies the local electronic structure and increases the chemical reactivity of carbon materials by creating unsaturated carbon atoms that can serve as catalytically active sites. In addition, defects modify the adsorption behavior of reactants and reaction intermediates, thereby facilitating charge transfer and improving reaction kinetics. Depending on the plasma conditions, defect engineering may also increase the specific surface area and expose additional active sites through controlled etching of the carbon surface [104,124].

However, the concentration of defects must be carefully controlled, as excessive defect generation may disrupt the graphitic structure, reduce electrical conductivity, and diminish the material’s structural stability. Thus, defect engineering requires balancing surface activation with preservation of graphitic ordering [104].

#### 4.1.3. Surface Functionalization

Plasma treatment enables the introduction of various functional groups onto the carbon surface, most commonly oxygen-containing species (e.g., hydroxyl, carbonyl, carboxyl, and epoxy groups) [36,61,115] whose type and concentration can be systematically tuned by adjusting plasma gas composition and operating conditions [27].

The incorporation of surface functional groups increases the surface energy and wettability of carbon materials, thereby improving the interaction between the electrode and the electrolyte and facilitating the transport of reactants. Furthermore, surface functionalities serve as anchoring sites for metal and metal oxide nanoparticles, promoting their homogeneous dispersion and enhancing catalyst stability. Appropriate control over the type and concentration of surface functional groups also enables tuning of the adsorption properties of carbon materials and their interactions with reactants during electrocatalytic processes [36,125,126,127,128].

#### 4.1.4. Synergistic Modification

In practice, plasma treatment often induces several modifications simultaneously, including heteroatom doping, defect generation, and surface functionalization. The coexistence of these modifications enables simultaneous tuning of the electronic structure, defect density, and surface chemistry of carbon materials. As a result, synergistically modified carbon materials frequently exhibit superior electrocatalytic performance compared with materials modified using a single strategy. The combined effects improve the density of active sites, optimize the adsorption of reaction intermediates, and facilitate charge-transfer processes [127,129].

Active-site assignments in heteroatom-doped carbons should nevertheless be treated cautiously. Correlations between catalytic performance and species identified by XPS, Raman spectroscopy, or theoretical calculations do not by themselves demonstrate that a particular heteroatom configuration constitutes the active site. Model-catalyst studies indicate, for example, that pyridinic nitrogen may create ORR-active Lewis-basic carbon atoms adjacent to the nitrogen atom, rather than acting directly as the adsorption site itself [130]. More generally, activity may originate from neighboring carbon atoms, coupled defect–dopant motifs, edge structures, trace metal species, or reconstructed sites formed under operating conditions. Under anodic OER conditions, carbon corrosion, surface reconstruction, and heteroatom leaching may further alter the initially characterized catalyst. Lu et al. demonstrated nearly complete dissolution of N, P, and Se dopants from heteroatom-doped carbon materials during water electrooxidation, with the resulting oxygen-rich residues and ortho-quinone moieties contributing to the observed OER activity [131]. Consequently, post-reaction and operando characterization are required to distinguish the as-prepared material from the catalytically relevant working state.

### 4.2. Applications in Electrocatalytic Reactions

Electrocatalytic reactions represent a major application area for plasma-modified carbon materials. Surface alterations introduced by plasma treatment—including defect formation, heteroatom configurations, and functional groups—directly shape the activity, selectivity, and stability of carbon-based electrocatalysts. In this subsection, recent representative studies are compared with respect to plasma-processing conditions, resulting physicochemical modifications, and electrocatalytic performance. The studies were selected as representative peer-reviewed examples providing sufficient information on both plasma-processing conditions and electrocatalytic performance. Quantitative tabular comparisons are provided for ORR, OER, and HER, whereas NRR and CO_2_RR are discussed descriptively because the available evidence remains limited.

This narrative review was based on peer-reviewed publications identified through searches of Web of Science, Scopus, and Google Scholar using combinations of the terms “plasma”, “carbon material”, “functionalization”, “electrocatalysis”, “oxygen reduction”, “oxygen evolution”, “hydrogen evolution”, “nitrogen reduction”, and “carbon dioxide reduction”. The literature search was last updated in August 2026. Studies were selected for quantitative comparison when plasma processing was directly involved in carbon modification, carbon-containing catalyst synthesis, or the treatment of a carbon-supported electrode architecture, and when sufficient plasma-processing or electrochemical data were available. Studies in which carbon served only as an unmodified conductive additive were excluded. All numerical values were verified against the original publications, and parameters not explicitly stated by the original authors are reported as “Not reported”.

Direct numerical comparison between the studies should be treated cautiously because catalyst composition, loading, electrode architecture, electrolyte, potential scale, current-density benchmark, iR-correction procedure, and stability protocol differ substantially. The tabulated values are therefore used primarily to identify processing–structure–performance relationships rather than to establish an absolute ranking of plasma-treated catalysts.

#### 4.2.1. Oxygen Reduction Reaction

Table 2 outlines the principal plasma-processing parameters used to modify carbon materials. Table 3 presents the electrochemical testing conditions and the ORR performance indicators for the synthesized electrocatalysts.

Among the systems for which *E*_1/2_ vs. RHE was reported, plasma-etched Fe@NCNT exhibited the highest half-wave potential, reaching 0.889 V. This performance suggests that plasma etching can improve the accessibility and electronic properties of existing Fe-N-C-type active sites without requiring extensive reconstruction of the catalyst. The N, P-codoped carbon-encapsulated Pt catalyst also shows favorable four-electron selectivity, low peroxide production, and high current retention, although its half-wave potential of 0.788 V is lower.

The remaining metal-free or multimetallic systems also predominantly approach a four-electron pathway, but their reported potentials are not always provided on the same reference scale. Several studies report onset or peak potentials relative to Ag/AgCl rather than *E*_1/2_ versus RHE, thereby preventing reliable ranking of activity. Moreover, differences in catalyst loading, carbon-black addition, rotation rate, and ink composition further limit direct quantitative comparison. Overall, the data support a mechanistic conclusion rather than a universal numerical ranking: ORR performance benefits when plasma processing combines accessible defects with appropriate heteroatom or metal–nitrogen coordination, provided that excessive disorder does not compromise conductivity.

The ORR studies encompass two distinct plasma strategies: post-synthesis etching of an existing carbon electrocatalyst and plasma-assisted synthesis of new carbon architectures. Direct nitrogen or argon plasma etching of Fe@NCNT primarily modifies the defect structure and surface chemistry of an already formed catalyst. In contrast, solution-plasma processing produces oxygen-rich, N/S-codoped, or N/P-codoped carbon materials directly from liquid precursors and may simultaneously determine carbon formation, heteroatom incorporation, defect generation, and nanoparticle encapsulation. The DBD treatment applied before MOF growth represents an intermediate strategy in which plasma activation modifies the carbonaceous precursor and affects the subsequent formation of the multimetallic catalyst architecture.

The available studies, therefore, do not identify a single optimal plasma configuration. Instead, they indicate that ORR enhancement is most consistently associated with the combined formation of structural defects and electronically active heteroatom environments. Nitrogen-containing sites contribute to oxygen adsorption and charge redistribution, while defects and edge sites increase the number of accessible reaction centers. In solution-plasma systems, however, plasma parameters cannot be interpreted independently of precursor composition and any subsequent thermal treatment. For example, the final properties of the N, P-codoped carbon-encapsulated Pt catalyst result from both plasma synthesis and annealing at 900 °C. Consequently, direct comparison of discharge voltage, frequency, and treatment time across gas-phase and liquid-phase plasma systems is not physically meaningful without corresponding information on plasma chemistry, energy deposition, and the resulting surface composition.

#### 4.2.2. Oxygen Evolution Reaction

Table 4 summarizes the key plasma-processing parameters used to modify carbon materials, whereas Table 5 presents the plasma-induced physicochemical changes and the corresponding electrocatalytic performance parameters for the oxygen evolution reaction.

OER results indicate improvements after plasma treatment, but the extent of improvement varies considerably. For Co(OH)_2_/GNP composites, oxygen-plasma modification leads to overpotentials in the approximate range of 310–390 mV at 10 mA cm^−2^ and Tafel slopes of 48–63 mV dec^−1^. The carbon@Co_3_O_4_ system reportedly shows an approximately 120 mV decrease in overpotential, accompanied by a Tafel slope near 50 mV dec^−1^. These examples support the conclusion that plasma-induced oxygen functionalities and interfacial restructuring can improve the dispersion, accessibility, and charge transfer of the active phase in carbon-supported cobalt catalysts.

The atmospheric-pressure plasma-treated systems show smaller or more system-dependent improvements. For NiFe/carbon paper, the overpotential at 100 mA cm^−2^ decreases only from 520.2 to 510.5 mV, while the Tafel slope decreases from 82.4 to 75.9 mV dec^−1^. This indicates a measurable but moderate kinetic benefit. Among the studies providing paired data, APPJ treatment of NiCo-MOF grown on carbon paper produced one of the largest reported improvements, decreasing the OER overpotential from 843 to 680 mV at 100 mA cm^−2^, corresponding to an absolute reduction of 163 mV, or approximately 19% [137]. For NiMoO_4_ grown on carbon paper, the optimal 60 s APPJ treatment yielded overpotentials of 368 mV at 10 mA cm^−2^ and 790 mV at 100 mA cm^−2^ [138]. These results demonstrate measurable improvements after short APPJ treatment, but direct comparison remains limited because the systems differ in catalyst composition and the reported current-density benchmarks.

**Table 4:** Quantitative Plasma Processing Conditions and Material Characterization for OER.

Study	Plasma Configuration	Exposure Method	Gas Atmosphere	Working Pressure	Plasma Power	Treatment Duration	Material
**Darvishzad & Stelmachowski (2025) [128]**	Low-temperature plasma	Direct plasma exposure of GNP powder	O_2_	0.2 mbar	100 W	5 min	Graphene nanoplatelets (GNP)
**Chueh et al. (2024) [138]**	Atmospheric-pressure plasma jet	Direct APPJ exposure	N_2_	Atmospheric pressure	4–50 W	30–90 s	NiMoO_4_/CP (hydrothermal synthesis)
**Yu et al. (2024) [139]**	Atmospheric-pressure plasma jet	Direct plasma surface modification of electrodeposited NiFe/CP	N_2_ + air	Atmospheric pressure	~700 W	60 s	NiFe/CP
**Pu et al. (2025) [137]**	Atmospheric-pressure plasma jet	Direct APPJ exposure on NiCo-MOF/CP	Ar	Atmospheric pressure	Not reported	60 s	NiCo-MOF grown on carbon paper
**Lofek et al. (2026) [126]**	Low-temperature plasma	Direct oxygen plasma exposure	O_2_	0.2 mbar	100 W	10 min	Carbon@Co_3_O_4_ nanoparticles

**Table 5:** Comparative Electrochemical OER Performance and Electrocatalytic Characteristics.

Study	Electrolyte	pH	Catalyst/Electrode Material	Electrode Configuration	iR-Correction	Overpotential (η at × mA cm^−2^)	Tafel Slope
**Darvishzad & Stelmachowski (2025) [128]**	1.0 M KOH	≈14	Co(OH)_2_/GNP composite on GC	Three-electrode (GC working, Pt counter, Ag/AgCl reference)	Applied	310–390 mV at 10 mA cm^−2^	48–63 mV dec^−1^
**Chueh et al. (2024) [138]**	1.0 M KOH	≈14	NiMoO_4_/CP (hydrothermal) + APPJ 60 s	Three-electrode (CP working, Pt counter, Ag/AgCl reference)	Applied	368 mV at 10 mA cm^−2^; 790 mV at 100 mA cm^−2^	109 mV dec^−1^
**Yu et al. (2024) [139]**	1.0 M KOH	≈14	NiFe/CP (as-deposited) and NiFe/CP-APP60	Three-electrode (CP working, Pt counter, Ag/AgCl reference)	Applied	Untreated: 520.2 mV; APPJ-treated: 510.5 mV, at 100 mA cm^−2^	Untreated: 82.4 mV dec^−1^; APPJ-treated: 75.9 mV dec^−1^
**Pu et al. (2025) [137]**	1.0 M KOH	≈14	NiCo-MOF grown on carbon paper; untreated and APPJ-treated for 60 s	Three-electrode	Applied	843 mV untreated; 680 mV after 60 s APPJ treatment, at 100 mA cm^−2^	Not Reported
**Lofek et al. (2026) [126]**	1.0 M KOH	≈14	Co_3_O_4_@C (pristine) and O_2_-plasma-activated Co_3_O_4_@C	Three-electrode (GC working, Pt counter, Ag/AgCl reference)	Applied	120 mV decrease after plasma treatment at 10 mA cm^−2^	Plasma-treated sample ~50 mV dec^−1^

Comparisons among the studies must therefore remain qualitative. The data include measurements at 10, 30, and 100 mA cm^−2^, different active phases, half-cell and possibly electrolyzer-level configurations, and diverse electrode geometries. The results nevertheless suggest that OER enhancement is frequently associated with modification of the active-phase/carbon interface or generation of a more favourable surface state, rather than with an increase in total defect concentration alone.

The OER dataset contains two fundamentally different modes of plasma action. Low-pressure oxygen plasma directly modifies graphene nanoplatelets or carbon@Co_3_O_4_ particles, primarily through surface oxidation, defect generation, improved wettability, and modification of the catalyst-support interface. Atmospheric-pressure plasma jets instead treat complete metal-containing electrodes, including NiMoO_4_/carbon paper, NiFe/carbon paper, and NiCo-MOF/carbon paper. In these systems, the plasma may simultaneously modify the active phase, carbon support, interfacial structure, surface oxidation state, and electrode morphology.

Treatment durations are generally short, ranging from tens of seconds for atmospheric-pressure plasma jets to several minutes for low-pressure oxygen plasma. However, duration alone does not represent treatment severity. One atmospheric-pressure treatment reaches a substrate temperature of approximately 500 °C in 60 s, indicating that thermal restructuring may accompany plasma-induced chemical effects. Such processing should not be interpreted as purely non-thermal surface functionalization. Similarly, nominal generator power cannot be compared directly between low-pressure and atmospheric-pressure systems because the fraction of energy delivered to the sample depends on reactor geometry, pressure, plasma volume, gas flow, and sample position.

The most convincing examples of carbon-specific plasma engineering are the oxygen-plasma treatment of GNP and carbon@Co_3_O_4_. In the other cases, the observed effects should be described more broadly as plasma engineering of carbon-supported electrode architectures because modification of the metal-containing active phase may dominate the catalytic response.

#### 4.2.3. Hydrogen Evolution Reaction

Table 6 summarizes the key plasma-processing parameters used to modify carbon materials, whereas Table 7 presents the plasma-induced physicochemical changes and the corresponding electrocatalytic performance parameters for the hydrogen evolution reaction.

Among the tabulated systems, Pt nanoparticles supported on plasma-prepared N-doped CNTs exhibit the lowest overpotential at 10 mA cm^−2^, reaching 28 mV in alkaline electrolyte. This high activity cannot be attributed solely to nitrogen-doped carbon, as Pt is the primary HER-active phase. The role of plasma is more specifically to generate nitrogen-containing anchoring sites, improve Pt dispersion, and influence electronic interactions at the Pt-carbon interface. The MoS_2_@WO_3_-anchored graphene material displays broader pH applicability, reaching 78 mV in acidic and 94 mV in alkaline electrolyte, with Tafel slopes of 48 and 68 mV dec^−1^, respectively. This result indicates that plasma-assisted construction of a conductive hybrid interface can improve charge transfer and expose active MoS_2_ sites.

The HER studies demonstrate three different plasma roles. First, low-pressure plasma acts as a short post-treatment for NiMoO_4_ supported on carbon paper. Second, nitrogen ICP treatment modifies carbon nanotubes prior to Pt deposition, thereby directly creating a nitrogen-doped carbon support. Third, electrochemical plasma exfoliation simultaneously produces graphene and anchors tungsten oxide species, after which MoS_2_ is deposited on the resulting support.

The treatments differ greatly in energy delivery and physicochemical outcome. The low-pressure post-treatments use low nominal power and short exposure times and are likely to modify only near-surface composition, defects, and oxidation states. The ICP treatment of Multi-walled carbon nanotubes (MWCNTs) is substantially longer and more energetic, promoting nitrogen incorporation and creating anchoring environments for ultrasmall Pt nanoparticles. Electrochemical plasma exfoliation is a synthesis route rather than a conventional surface treatment, because it simultaneously generates the carbon structure and introduces the tungsten-containing component. The most effective plasma strategy depends on whether the objective is to activate an existing catalyst, engineer a carbon support for metal dispersion, or construct a new hybrid architecture.

#### 4.2.4. Emerging Applications: Nitrogen Reduction Reaction (NRR) and Carbon Dioxide Reduction Reaction (CO_2_RR)

Applications of plasma-engineered carbon-containing materials in NRR and CO_2_RR remain limited compared with ORR, OER, and HER. Nevertheless, the available studies demonstrate that plasma-assisted processing can generate low-coordinate sites and reconstruct local metal–nitrogen environments, thereby facilitating the adsorption and activation of stable N_2_ and CO_2_ molecules.

For NRR, Yang et al. used plasma-assisted calcination to prepare IrP_2_ nanocrystals embedded in phosphorus- and nitrogen-codoped porous carbon nanofilms. The formation of low-coordinate step sites enhanced nitrogen activation, yielding an NH_3_ production rate of 94.0 μg h^−1^ mg^−1^ and a maximum Faradaic efficiency of 17.8% [143]. Nevertheless, electrocatalytic NRR results require particularly rigorous validation because the low quantities of generated NH_3_ are susceptible to contamination from gases, electrolytes, catalysts, and laboratory environments. Reliable confirmation should therefore include appropriate Ar and open-circuit controls, analysis of potential nitrogen-containing contaminants, and isotope-labelling experiments using purified ^15N_2_, with independent detection of ^15^NH_3_. Commercial ^15^N_2_ itself may contain ^15^NH_3_ and oxidized ^15^N impurities at levels comparable to reported NRR yields, making purification and contamination testing of the isotope feed essential [144,145].

For CO_2_RR, Jia et al. used a domestic microwave oven to generate low-pressure plasma in a round-bottom flask, enabling continuous plasma treatment that reconstructed pyrrolic-N defects surrounding isolated Ni sites into a pyridinic-N-dominated Ni–N_2_ coordination environment. The reconstructed catalyst reached a CO Faradaic efficiency of 96% at an overpotential of 590 mV and a CO current density of 33 mA cm^−2^ at an overpotential of 890 mV [146].

These studies indicate that plasma processing can influence electrocatalytic performance not only by increasing the overall defect density but also by creating specific low-coordinate sites and reconstructing local metal–nitrogen coordination environments. However, the limited number of reports does not yet permit general relationships among plasma conditions, active-site structure, and NRR or CO_2_RR performance to be established.

#### 4.2.5. Summary of Electrocatalytic Applications

Comparison of the ORR, OER, and HER studies shows that plasma processing does not act through a single universal mechanism. For ORR, its principal role is the combined engineering of carbon defects, heteroatom environments, and metal–nitrogen coordination. For OER, the dominant benefit is often attributed to modification of the carbon surface and the catalyst-support interface, although plasma-induced restructuring of the active phase may also contribute. For HER, plasma treatment is most effective for improving active-phase dispersion, anchoring, and interfacial charge transfer rather than for creating intrinsically active carbon sites. Across all three reactions, nominal plasma power and treatment time alone are insufficient descriptors of processing severity. Meaningful structure-activity relationships require simultaneous reporting of plasma configuration, pressure, gas composition, sample temperature, treatment geometry, post-plasma exposure conditions, and the resulting surface composition. Electrocatalytic comparisons should likewise prioritize improvements relative to appropriate untreated controls because differences in catalyst composition, loading, electrode architecture, electrolyte, current-density benchmark, and iR correction prevent reliable ranking based solely on absolute performance values.

## 5. Advantages, Limitations, and Scalability

### 5.1. Advantages

Plasma modification is a rapid and versatile technique for the functionalization of carbon materials. Unlike conventional wet-chemical methods, it does not require large amounts of solvents or aggressive reagents and can often be performed at relatively low temperatures. In addition, treatment times typically range from a few seconds to several minutes, making plasma processing an attractive alternative for surface modification [28].

Another important advantage of plasma processing is the possibility of systematically adjusting gas composition, power, pressure, and treatment time to tune the resulting surface chemistry, wettability, and defect density. This enables tailoring of the surface chemistry, wettability, and defect density of carbon materials, allowing material properties to be adjusted under appropriately controlled conditions. Moreover, atmospheric-pressure and roll-to-roll plasma systems show promising potential for continuous processing. However, further studies, including life-cycle and techno-economic analyses, are needed to fully assess the environmental, economic, and industrial viability of plasma-based technologies.

### 5.2. Limitations and Challenges

Despite the many advantages of plasma modification, the technique is associated with several limitations. Due to the surface-specific nature of plasma-material interactions, the induced changes occur primarily within the outermost layers of the material, which may limit the effectiveness of modification in thick or highly porous structures. Excessive powder bed thickness during plasma treatment leads to a shielding effect, in which only the topmost particle layers are effectively functionalized, while the bulk of the powder remains largely unmodified; this highlights the need for thin, well-dispersed powder layers (or agitation/fluidization) to ensure homogeneous plasma treatment across the entire sample. In addition, inappropriate process parameters, such as excessive plasma power or prolonged exposure time, can lead to over-etching of the surface, an increased defect density, and deterioration of the material’s electrical properties [28].

Another significant challenge is the lack of standardization in reporting plasma treatment parameters, which complicates comparisons of results across studies. Furthermore, the complex nature of plasma makes identifying the mechanisms responsible for surface modification highly demanding and often requires advanced diagnostic techniques. As a result, a comprehensive understanding of the relationship between process parameters and material properties remains an active area of research.

### 5.3. Scalability and Industrial Prospects

Although plasma modification of carbon materials is currently applied predominantly at the laboratory scale, the technology has attracted increasing interest for potential industrial implementation. In particular, atmospheric-pressure plasma systems eliminate the need for vacuum equipment and may facilitate continuous processing, making them attractive for integration with manufacturing technologies such as roll-to-roll production of carbon films, conductive textiles, flexible electrodes, and other carbon-based materials.

Additionally, plasma treatment may offer an alternative to conventional multi-step chemical functionalization approaches. Nevertheless, further efforts are required to establish standardized operating protocols, quality-control procedures, and reproducible processing conditions for large-scale manufacturing. Moreover, the environmental impact, energy requirements, economic feasibility, and scalability of plasma-based processes should be further evaluated through comprehensive life-cycle and techno-economic analyses. Overall, plasma technologies show promising potential for the production of advanced carbon-based materials, although further studies are needed to verify their industrial viability.

## 6. Conclusions and Outlook

Plasma-based modification has emerged as a versatile approach for tailoring the physicochemical properties of carbon materials used in electrocatalytic applications. The studies reviewed in this work consistently demonstrate that plasma treatment can effectively modify surface chemistry, generate structural defects, and introduce heteroatom functionalities, often concentrating these changes in the near-surface region. Across the investigated electrocatalytic reactions, the most reproducible effects of plasma processing include increased defect density, enhanced heteroatom incorporation, improved surface wettability, and greater accessibility of catalytically active sites. These modifications are frequently associated with improved ORR, OER, and HER performance, whereas studies on NRR and CO_2_RR remain comparatively limited. Despite the growing number of reports, several mechanistic aspects remain insufficiently understood. In particular, the relationship between plasma operating conditions, the nature of plasma-generated active sites, and the resulting electrocatalytic performance is often difficult to establish due to differences in plasma configurations, treatment parameters, and characterization protocols. The lack of standardized reporting practices further complicates direct comparison between studies.

On the basis of the available evidence, future plasma-electrocatalysis studies should report, at minimum, the plasma configuration, direct or remote exposure mode, gas composition and flow, pressure, electrical excitation conditions, treatment time, substrate temperature, sample geometry or mass, and post-plasma handling time. These parameters should be accompanied by quantitative characterization of surface composition, defect structure, porosity, wettability, conductivity, and active-phase dispersion, together with appropriately matched untreated controls and complete electrochemical testing conditions. Such reporting is essential for determining when plasma treatment offers a reproducible advantage over conventional functionalization.

## Figures and Tables

**Table 1:** Classification of plasma systems and schematic overview of reactive species and plasma–surface interaction mechanisms.

	Low-Pressure Plasmas	Atmospheric-Pressure Plasma
**Systems**	RF, Microwave, ICP	DBD, Plasma jets
**Key features**	High controllability; high density; controlled ion energy	Vacuum-free operation; scalable processing; localized or large-area treatment
**Reactive species**	Electrons; ions; radicals; metastables; UV photons	Radicals; ions; metastables; UV photons
**Dominant mechanisms**	Ion bombardment; bond scission; sputtering; functionalization	Radical-driven reactions; surface activation; cleaning; functionalization
**Typical effects**	Defect generation; surface functionalization; etching	Surface activation; functionalization; etching

**Table 2:** Quantitative Plasma Processing Conditions and Material Characterization for ORR.

Study	Plasma Configuration	Exposure Method	Gas Atmosphere	Plasma Phase	Working Pressure	Plasma Power/Voltage	Treatment Duration	Material
**Rao et al. (2024) [132]**	RF plasma etching (13.56 MHz)	Direct plasma etching of Fe@NCNT powder	N_2_ or Ar	Gas-phase plasma	Not reported	Not reported	Not reported	Fe@NCNT-PN (N_2_ plasma) and Fe@NCNT-PAr (Ar plasma)
**Kim et al. (2023) [133]**	Solution plasma process (SPP), bipolar pulsed discharge	Direct plasma discharge in liquid benzene + crown ether	Reactive species from liquid precursors	Liquid-phase plasma	Atmospheric pressure	1.2 kV bipolar pulsed power supply (pulse width 0.8 µs, 25 kHz)	10 min	Oxygen-rich defect nanocarbon
**Zhao et al. (2024) [134]**	Dielectric barrier discharge (DBD)	Surface treatment of PPy nanotubes before MOF growth	Ambient air	Gas-phase plasma	Atmospheric pressure	75 V (DBD discharge voltage)	5 min	CoNiFe alloy nanoparticles in N-doped carbon nanosheets on N-CNT networks
**Hyun & Chae (2025) [135]**	Solution plasma process (SPP), bipolar pulsed discharge	Plasma discharge in liquid precursor (2-pyrrolidone + γ-thiobutyrolactone)	Reactive species from liquid precursors	Liquid-phase plasma	Atmospheric pressure	200 kHz bipolar pulsed discharge; applied voltage not reported	Not reported	N, S-doped carbon nanosheets (NSCNs)
**Ukai et al. (2025) [136]**	Solution plasma process (SPP), bipolar DC pulses	Plasma generated directly in liquid between carbon electrodes	Reactive species from liquid precursors	Liquid-phase plasma	Atmospheric pressure	1 kV bipolar DC pulses (50–100 kHz)	5 min	N, P-doped carbon encapsulating Pt nanoparticles

**Table 3:** Comparative Electrochemical ORR Performance and Electrocatalytic Characteristics.

Study	Electrolyte	pH	Catalyst Layer Preparation	Electrode Configuration	iR-Correction	Half-Wave Potential (E_1/2_)	ORR Activity Characteristics	Tafel Slope	Stability Assessment
**Rao et al. (2024) [132]**	0.1 M KOH	≈13	Catalyst powder dispersed in standard RDE ink (not explicitly described)	Three-electrode system: GC-RDE (working), Pt wire (counter), Ag/AgCl (reference)	Not reported	0.889 V (vs. RHE)	Kinetic current density: 3.064 mA cm^−2^ at 0.90 V (RHE); 4-electron ORR pathway	Not reported	20,000 cycles ADT; 93.7% retention after 24,000 s CA
**Kim et al. (2023) [133]**	0.1 M KOH	≈13	5 mg catalyst + 0.5 mL ethanol + 50 µL 5 wt% Nafion; sonicated 30 min; 20 µL ink drop-cast on GC electrode	Three-electrode system: GC-RDE (working), Pt wire (counter), Ag/AgCl (reference)	Not reported	Not reported	ORR onset: −0.154 V (vs. Ag/AgCl); ORR peak: −0.254 V; electron transfer number *n* ≈ 3.5 (CNP-CE-20); highest limiting current for CNP-CE-50	Not reported	Not reported
**Zhao et al. (2024) [134]**	0.1 M KOH	≈13	Catalyst ink prepared on GC-RDE; loading not explicitly reported	Three-electrode system: GC-RDE (working), Pt plate (counter), saturated Hg/HgCl_2_ (reference)	Not reported	Not reported	Limiting current density: 5.73 mA cm^−2^ at 1600 rpm; electron transfer number *n* ≈ 4 (RRDE); low HO_2_^-^ yield; first-order O_2_ kinetics (K-L plots)	Not reported for ORR in the article	Chronoamperometry at −0.2 V (vs. SCE) for 10,000 s; high current retention vs. Pt/C
**Hyun & Chae (2025) [135]**	0.1 M KOH	≈13	1 mg NSCNs + 4 mg carbon black + ethanol/water + 40 µL Nafion; sonicated; 10 µL drop-cast on 4 mm GC (loading 0.4 mg cm^−2^)	Three-electrode system: GC-RDE (working), Pt wire (counter), Ag/AgCl (reference)	Not reported	Not reported	ORR onset: −0.135 V (vs. Ag/AgCl); cathodic peak: −0.33 V; electron transfer number *n* ≈ 4 at potentials < −0.43 V; limiting current increases with rpm (500–2500 rpm)	Not reported	Chronoamperometry at −0.4 V for 45,000 s; NSCNs retain ~95% current; Pt/C retains ~71%
**Ukai et al. (2025) [136]**	0.1 M KOH	≈13	5 mg catalyst + 500 µL ethanol + 50 µL Nafion; sonicated 30 min; 3 µL drop-cast on 4 mm GC (CV) and 5 µL on RRDE disk; dried 6 h	RRDE: GC disk (4 mm) + Pt ring; Pt coil counter; Ag/AgCl (sat. KCl) reference; potentials converted to RHE	Not reported	0.788 V (vs. RHE)	Limiting current density: 5.28 mA cm^−2^ at 1600 rpm; onset potential: 0.838 V; electron transfer number *n* ≈ 4.0; H_2_O_2_ yield < 6%	Not reported	Chronoamperometry at −0.4 V for 45,000 s; 94% current retention

**Table 6:** Quantitative Plasma Processing Conditions and Material Characterization for HER.

Study	Plasma Configuration	Exposure Method	Gas Atmosphere	Working Pressure	Plasma Power	Treatment Duration	Material
**Chueh et al. (2025) [140]**	Low-pressure RF plasma	Direct plasma treatment of NiMoO_4_/CP	N_2_	6 × 10^−2^ Torr	11 W	30–90 s	NiMoO_4_ nanowires on carbon paper (NiMoO_4_/CP)
**Zhao et al. (2024) [141]**	RF inductively coupled plasma (ICP)	Plasma N-doping of MWCNTs	N_2_	Low-pressure (ICP; NR)	250 W	20 min	N-doped MWCNTs supporting Pt nanoparticles (Pt/NCNTs)
**Dang et al. (2024) [142]**	Electrochemical plasma exfoliation	Plasma-induced exfoliation of graphite + anchoring WO_3_	Electrolyte plasma (H_2_SO_4_)	Not applicable, plasma generated at the electrode–electrolyte interface	65 V (plasma generated electrochemically)	30–180 min	WO_3_-anchored graphene nanosheets (W-G)

**Table 7:** Comparative Electrochemical HER Performance and Electrocatalytic Characteristics.

Study	Electrolyte	pH	Catalyst/Electrode Material	Electrode Configuration	iR-Correction	Overpotential (η at × mA cm^−2^)	Tafel Slope
**Chueh et al. (2025) [140]**	1 M KOH	≈14	NiMoO_4_/CP (hydrothermal growth)	Three-electrode NiMoO_4_/CP working, GC counter, Ag/AgCl reference)	Applied	289 mV at 10 mA cm^−2^	222 mV dec^−1^
**Zhao et al. (2024) [141]**	1.0 M KOH	≈14	Pt/NCNTs (catalyst ink on GCE)	Three-electrode (GCE working, graphite counter, Hg/HgO reference)	Not reported	28 mV at 10 mA cm^−2^	53 mV dec^−1^
**Dang et al. (2024) [142]**	0.5 M H_2_SO_4_ (acidic) and 1 M KOH (alkaline)	Acidic: ~0.3; Alkaline: ~14	MoS_2_@W-G	Three-electrode (MoS_2_@W–G working, Pt counter, RHE/Ag/AgCl reference)	Not reported	78 mV (acidic) at 10 mA cm^−2^; 94 mV (alkaline) at 10 mA cm^−2^	48 mV dec^−1^ (acidic); 68 mV dec^−1^ (alkaline)

## Data Availability

No new data were created or analyzed in this study. Data sharing is not applicable to this article.
